# A Machine Learning Approach for Path Loss Prediction Using Combination of Regression and Classification Models

**DOI:** 10.3390/s24175855

**Published:** 2024-09-09

**Authors:** Ilia Iliev, Yuliyan Velchev, Peter Z. Petkov, Boncho Bonev, Georgi Iliev, Ivaylo Nachev

**Affiliations:** Department of Radio Communications and Video Technology, Faculty of Telecommunications, Technical University of Sofia, 1000 Sofia, Bulgaria; igiliev@tu-sofia.bg (I.I.); julian_s_velchev@tu-sofia.bg (Y.V.); bbonev@tu-sofia.bg (B.B.); simbata1234@gmail.com (G.I.); ivaylonachev@tu-sofia.bg (I.N.)

**Keywords:** path loss prediction, radio propagation modeling, LoRa, neural network regression, neural network classification

## Abstract

One of the key parameters in radio link planning is the propagation path loss. Most of the existing methods for its prediction are not characterized by a good balance between accuracy, generality, and low computational complexity. To address this problem, a machine learning approach for path loss prediction is presented in this study. The novelty is the proposal of a compound model, which consists of two regression models and one classifier. The first regression model is adequate when a line-of-sight scenario is fulfilled in radio wave propagation, whereas the second one is appropriate for non-line-of-sight conditions. The classification model is intended to provide a probabilistic output, through which the outputs of the regression models are combined. The number of used input parameters is only five. They are related to the distance, the antenna heights, and the statistics of the terrain profile and line-of-sight obstacles. The proposed approach allows creation of a generalized model that is valid for various types of areas and terrains, different antenna heights, and line-of-sight and non line-of-sight propagation conditions. An experimental dataset is provided by measurements for a variety of relief types (flat, hilly, mountain, and foothill) and for rural, urban, and suburban areas. The experimental results show an excellent performances in terms of a root mean square error of a prediction as low as 7.3 dB and a coefficient of determination as high as 0.702. Although the study covers only one operating frequency of 433 MHz, the proposed model can be trained and applied for any frequency in the decimeter wavelength range. The main reason for the choice of such an operating frequency is because it falls within the range in which many wireless systems of different types are operating. These include Internet of Things (IoT), machine-to-machine (M2M) mesh radio networks, power efficient communication over long distances such as Low-Power Wide-Area Network (LPWAN)—LoRa, etc.

## 1. Introduction

In recent years, radio access networks have developed at an unprecedented pace. They are generally the bottleneck in end-to-end network communications due to a number of natural phenomena in the propagation of electromagnetic waves, especially in mobile communications. The communication channel is dispersive in time and frequency. The radio interface is exposed to noise and multiple interference of an intra- and inter-system nature. On the other hand, with the evolution of new-generation cellular networks, the emergence of the Internet of Things (IoT) and the Internet of Everything (IoE) has not only expanded the application of wireless networks, but has necessitated the flexible use and reuse of the radio spectrum. Information transmission speeds, and the number and type of telecommunications services are steadily increasing. These changing conditions have entailed new approaches to radio interface construction, in terms of radio signals’ synthesis, modulation, and coding, and continuous adaptation of the radio link and its parameters to the propagation environment. The emergence of software-defined radio has enabled the construction of cognitive radio networks, at the core of which is a cognitive machine. The current trend is to implement cognitive radio using artificial intelligence to maximize the throughput of the communication channel under certain conditions of the electromagnetic wave propagation environment.

In radio communications, all elements of machine learning (ML) and self-learning are applicable due to the continuously changing radio communication environment and communication channel conditions. A key element of the cognitive process is adapting the relationship of the interrelated information and energy parameters of the channel. In order to provide a certain type of communication service, it is necessary to maintain within given limits the basic quality parameters, such as bandwidth, data rate, signal-to-noise interference ratio, binary error rate, etc.

The radio link power budget is used in two aspects. The first one is preliminary engineering planning of the the radio link. The second is the process of its use and adaptation by radio communication facilities. These processes are part of the functions of the physical, media access control (MAC) and radio link control (RLC) layers of the communication protocols. For example, they are an important element in interference reduction in cellular networks through transmitter power management procedures.

The radio link power budget requires the knowledge or prediction of electromagnetic wave propagation losses. Propagation loss models allow us to determine the received signal power as a function of distance and other parameters, and hence to predict the signal-to-noise ratio at the receivers. Given the distance and other parameters of the radio communication system, the terrain, and its characteristics and propagation conditions, the maximum allowable path loss of the radio communication can be determined. Alternatively, given the attenuation, the maximum radius of radio coverage can be calculated.

The losses are determined by several natural phenomena in the propagation of an electromagnetic wave, such as decrease in power density of the electromagnetic wave as a function of distance, diffraction, reflection, and scattering. In most cases, this necessitates the use of a multi-path propagation model, and, in the case of mobility, it is necessary to account for the appearance of fading and Doppler shift. Propagation models may include the influence of large-scale fading. Small-scale fading and Doppler shift are not included in the models because their influence is minimized in the signal processing stage at the transmitting or receiving side. On this basis, a number of models have emerged, which can be divided into three main groups: deterministic (analytical); stochastic; and empirical.

The deterministic models use exact analytical expressions derived from electrodynamics and are directly related to the propagation environment and the characteristics of the region of coverage. They use a multi-path propagation model and accurate 2D or 3D maps of the terrain with detailed electromagnetic characteristics of the obstacles. Many of them apply finite-difference time-domain methods for attenuation estimation [1,2]. Their accuracy is relatively high, but the computational complexity is significant. A classic representative of theses types of models is the Longley–Rice model [3].

The stochastic models account for the random nature of the large-scale fading, which is described by a log-normal distribution. Based on statistical parameters and a given coverage probability, an additional large-scale fading margin is included [4,5]. Based on a number of studies, the 3rd Generation Partnership Project (3GPP) organization proposes TR 38.901 stochastic empirical models for 5G NR for the frequency range 0.5–100 GHz [6]. Models for different coverage scenarios are synthesized, such as Rural Macro, Urban Macro, Urban Micro, Indoor Factory, Indoor Hotspot, etc.

Empirical models are obtained under certain conditions after approximation of a set of measured data. They are characterized by their simplicity and relatively high modeling accuracy, but only for the conditions under which they are defined. Over time, several classical models have been established and used for coverage prediction in radio communication systems: Okumura–Hata model [7], COST 231 Hata [8], COST Walfisch–Ikegami [9], Lee model [10], etc. For example, in the Lee model, a simplified loss model is applied to predict the attenuation as a function of distance. Additionally, correction factors for antenna heights, operating frequency, and antenna gains are included.

A current trend in radio coverage prediction is by using ML models trained with supervised, unsupervised, and reinforcement learning algorithms [11,12,13,14,15]. The application of ML in the models not only enhances the accuracy of coverage prediction, but also allows their direct implementation in the cognitive machine in cognitive radio networks in order to adapt the link, depending on the communication environment conditions.

Supervised learning algorithms find a special place in loss modeling because the task involves both classification and regression elements. The modeling of propagation losses is in most of the cases interpreted as a regression problem. Support vector machines, Gaussian process regression models, kernel approximation and classifiers, neural networks, naive Bayes classifiers, nearest neighbor classifiers, random forest ensemble learning algorithms, etc., are applied. As an example, a number of publications have not only performed comparative analysis, but also demonstrated multiple studies on the parameters of propagation loss models using ML algorithms [11,16,17,18,19,20,21].

In [16], a neural network ensemble learning technique with increased accuracy for path loss prediction is proposed. The measured results are taken from [22], which are for an 1800 MHz frequency band in urban area propagation conditions. The neural network uses six input parameters (features): longitude; latitude; elevation; altitude; clutter height; and distance. Some of the used indicators of the model’s performance are root mean square error, RMSE [17]; mean absolute error, MAE [18]; and coefficient of determination, $R^{2}$ [16]. The achieved results are as follows: RMSE=2.941 dB; MAE=1.2753 dB; and $R^{2}=0.8951$.

Paper [17] proposes support vector regression (SVR) and radial basis function (RBF) models for path loss predictions in rural, suburban, and urban areas. The following environmental input parameters are used: elevation; clutter heights; distance; altitude; building-to-building distance; the street orientation angle; and base station antenna heights (fixed to 25 m and 35 m). The obtained RMSE values for the three types of areas are 1.378 dB; 1.452 dB; and 2.157 dB. The results show a very good regression fitting because the measured points are located approximately on three straight lines originating at the base station. Unfortunately, we did not find any information about the operating frequency.

A technique that combines artificial neural networks (ANNs) with Gaussian process (GP) variance analysis and principal component analysis is proposed in [11]. The multi-layer perceptron (MLP) is the core of the ANN architecture. The input parameters of the model are frequencies (450 MHz, 1450 MHz, and 2300 MHz); elevation plus transmitting antenna height; elevation plus receiving antenna height; and the difference between these two heights. The data are collected for only one transmitting antenna height (15 m) and for suburban propagation conditions (small town). The achieved quantities for the regression performance using the ANN for frequency 450 MHz are as follows: RMSE=7.876 dB; MAE=5.896 dB; and $R^{2}=0.3975$.

Three ML regression models are investigated and compared in [18]: ANN; support vector machine; and multi-linear regression. The measurements are made for one frequency (900 MHz) and one transmitter antenna height (100 m). The communication distance is limited within the range 100 m to 800 m, and a very few number of geographical points (<100) are considered. We believe this is the reason for the very good approximation results that are reported (*RMSE* = 0.008438 and $R^{2}$ = 0.999675) when using ANNs.

An ensemble method consisting of three neural network models—conventional ANNs, long short-term memory-based recurrent neural networks, and convolutional neural networks—is analyzed in [19]. The prediction of path loss is for an indoor environment at three frequencies of 14 GHz, 18 GHz, and 22 GHz. The data used in this research are collected in an indoor environment for line-of-sight (LOS) and non-line-of-sight (NLOS) scenarios. The input features are distance; frequency; angle of arrival; and transmitter antenna height. The distance ranges from 2 to 24 m. Measurements at various frequencies are carried out for 865 points. The studied models demonstrate high accuracy in terms of the maximum value of RMSE being less than 0.3162 dB and the average value of $R^{2}$ being as high as 0.9753.

Our analysis shows that many successful ML-based techniques for path loss prediction have been developed. In terms of their approximation accuracy, adequacy and generality, the choice of the method is of big concern. The proper selection of input parameters, training data size, and consideration of the propagation conditions of the electromagnetic wave are of key importance. Based on the physical processes of electromagnetic wave propagation, the most influential parameter is the distance between the transmitter and the receiver. The other parameters, which are related to the diffraction properties of the electromagnetic wave, are the heights of the antennas, the terrain profile, and the type and characteristics of the obstacles. The propagation medium influences the reflection and multi-path propagation, expressed as statistical parameters of the long-scale fading. Each different propagation area has specific dispersion. The operating frequency is also an important parameter because it directly influences the free space propagation losses. It also affects the additional attenuation caused by hydrometeors, vegetation, buildings, and air molecules. An additional influence on the accuracy of the approximation is the measurement error of the primary parameters. Most of the proposed models are limited by the conditions under which the training samples are obtained. They provide the relevant accuracy only under these conditions. For this reason, the comparison between different methods is sometimes incorrect.

In this study, a mobile measurement is performed using specially designed equipment and software. The values of the measured attenuation are for a frequency of 433 MHz. The main reason for the choice of such an operating frequency is because it falls into the industrial, scientific, and medical (ISM) range, in which the number and type of operating wireless systems are increasing. These include IoT, machine-to-machine (M2M) mesh radio networks, power efficient communication over long distances such as Low-Power Wide-Area Network (LPWAN)—LoRa, etc. IoT is the basis of a number of information telecommunication systems, such as Smart Homes, Smart Cities, Smart Grid, Cyber Physical Systems, and others. The use of these systems re-proposes different coverage scenarios, diversity of propagation environments, and different antenna heights, especially of the end devices that are close to low-altitude obstacles. These are the prerequisites for our motivation to face this problem.

## 2. The Proposed Approach

### 2.1. Overview

This study proposes and investigates a path loss prediction ANN-based approach using combination of regression and classification models. This compound model is adequate for rural, suburban, and urban areas. The novelty here is that an additional classifier is applied, through which the model automatically estimates the type of coverage scenario—LOS or NLOS, based on indirect input parameters.

**Figure 1:**
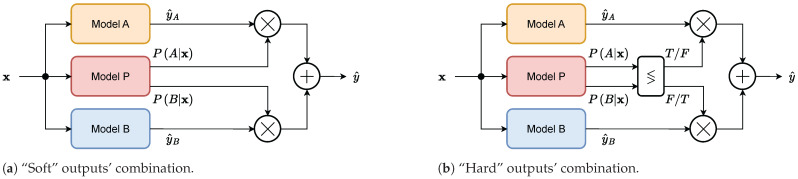
Architecture of the compound model for path loss prediction in two variants of combining the outputs of the two regression models (**a**,**b**).

The proposed compound model for path loss prediction is composed of two regression models (named Model A and Model B), whose outputs can be combined by different manners (Figure 1).

Model A is adequate in the LOS scenario, whereas Model B is suitable for NLOS conditions. A third model (named Model P), which shares the same input parameters as Models A and B, serves as a binary classifier. Its outputs can be considered as posterior probabilities $P\left(A|x\right)$ and $P\left(B|x\right)$ for the input data, x, being adequate to each regression model, where A is for the LOS scenario and B is for NLOS. The outputs ${\hat{y}}_{A}$ and ${\hat{y}}_{B}$ of the regression models can be combined as a weighted average using $P\left(A|x\right)$ and $P\left(B|x\right)$ as coefficients (Figure 1a). This can be expressed as follows:

(1) $$\hat{y}=P\left(A|x\right){\hat{y}}_{A}+P\left(B|x\right){\hat{y}}_{B},$$

where $\hat{y}$ is the output of the compound model. This type of combination is referred as “soft” in the rest of the paper. A simpler variant is shown in Figure 1b. The output of the compound model is just the output of a particular regression model considering the maximal probability among $P\left(A|x\right)$ and $P\left(B|x\right)$:

(2) $$\hat{y}=\left\{\begin{array}{cc} {\hat{y}}_{A}, & P\left(A|x\right)\geq P\left(B|x\right)\\ {\hat{y}}_{B}, & P\left(A|x\right)<P\left(B|x\right) \end{array}\right..$$

This type of combination is referred as “hard”. Any of these probabilities can be routed as an additional output, so this can serve as an uncertainty indicator for the selection of a particular regression model.

In order to perform the regression and classification with the required accuracy, the training of the neural networks is performed with an ensemble of measured data. It consists of terrain parameters for the three types of areas, type and height of the buildings, and antenna heights.

### 2.2. Input and Output Parameters

Since Model P has to be trained using supervised learning, true labels have to be provided. They are denoted as LOS and report whether the profile has a direct or indirect line of sight. This binary quantity is determined automatically by a procedure written in MATLAB©. The input parameters of the procedure are the coordinates of the measured point, the coordinates of the stationary station, and the antenna heights. The geographic data of terrain relief and 3D buildings for specified regions of interest taken from [23] are passed for 3D visualization and profile tracking analysis. The constructed altitude profile with obstacles between a measured point and the stationary station and the straight line between the two antennas’ heights determine whether there is a direct or indirect line of sight.

The model is proposed to have five input parameters: the 2D distance between the receiver and the transmitter in logarithmic scale, $d_{log}$; the relative height, $h_{eff}$, between the antennas of the stationary and mobile stations; the relative maximum terrain height, $H_{r,MAX}$, including obstacles; the relative average height of the terrain, $H_{r,AV}$; and the standard deviation of the profile of the propagation path, $H_{r,STD}$. The chosen input parameters can be expresses as a 5-dimensional vector:

(3) $$x=\left[\begin{array}{ccccc} x_{1} & x_{2} & x_{3} & x_{4} & x_{5} \end{array}\right].$$

The first element is the distance *d* (in meters) in logarithmic scale:

(4) $$x_{1}=d_{log}=20\log_{10}d.$$

The attenuation is a linear function of distance expressed in logarithmic scale.

The second element is the effective antenna height in meters:

(5) $$x_{2}=h_{eff}=\left|\left(h_{S}+A_{S}\right)-\left(h_{M}+A_{M}\right)\right|,$$

where $h_{S}$ and $h_{M}$ are the antenna heights relative to the terrain for the stationary and mobile transceivers, respectively. $A_{S}$ and $A_{M}$ are their corresponding altitudes. Instead of using fixed absolute values for antenna heights (as in many other models), the effective height here is the difference in the heights of the transmitter and receiver antennas, including altitudes of the installation positions. This choice extends the applicability of the model. This parameter is calculated as a absolute value because the link channel is assumed to be reversible concerning the transmitter–receiver direction.

The relative maximal height of the terrain, $H_{r,MAX}$, and obstacles in respect to antenna heights as well participate in the input parameters as the third element of x:

(6) $$x_{3}=H_{r,MAX}=\max_{\begin{array}{c} j=1..N_{k} \end{array}}\left(A_{j}\right)-\min\left\{\left(h_{S}+A_{S}\right),\left(h_{M}+A_{M}\right)\right\}+H_{B,AV},$$

where $A_{j}$ is the elevation at position *j* of the altitude profile between the measurement point and the stationary station. The altitude profile is divided into $N_{k}$ parts with a step Δd=6m, which is the half of a street’s width for a small town. The calculation is made relative to the lowest height of the two antennas in order to account for the worst case in terms of shadowing. The input parameter includes the average height, $H_{B,AV}$, of buildings for the area of consideration.

The fourth input parameter is the average height of the altitude, $H_{r,AV}$, in the direction transmitter–receiver in respect to the antenna heights:

(7) $$x_{4}=H_{r,AV}=\frac{1}{N_{k}}\sum_{j=1}^{N_{k}}A_{j}-\max\left\{\left(h_{S}+A_{S}\right),\left(h_{M}+A_{M}\right)\right\}.$$

The last input parameter is the standard deviation, $H_{r,STD}$, of the altitude profile in respect to the antenna heights:

(8) $$x_{5}=H_{r,STD}=\sqrt{\frac{1}{N_{k}-1}\sum_{j=1}^{N_{k}}{\left(A_{j}-\bar{A}\right)}^{2}}-\max\left\{\left(h_{S}+A_{S}\right),\left(h_{M}+A_{M_{k}}\right)\right\},$$

where $\bar{A}$ denotes the mean elevation value.

Finally, the x vector is a subject of linear transformation. The transformation is chosen so that when applied on the whole dataset matrix:

$$X=\left[\begin{array}{c} x_{1}\\ x_{2}\\ \vdots \\ x_{N} \end{array}\right],$$

the result will be a matrix whose columns have zero mean and unit variance. This data standardization enhances the performance of the models and improves the accuracy.

The input parameters are selected to help the model to account for the effects of electromagnetic wave diffraction and shadowing losses, and to distinguish between LOS and NLOS scenarios. The last three parameters ($x_{3}$, $x_{4}$, and $x_{5}$) are statistical quantities that refer to the path profile between transmitter and receiver. The parameter $H_{r,MAX}$ takes into account the highest point of the line transmitter–receiver and the average height, $H_{B,AV}$, of the buildings for the area. This leads to an increase in its value without describing the actual situation. There may or may not be a building at the highest point of the profile. The actual height is not involved here, but the average height for the area is used instead. At first glance, this would increase the regression error if the model had only a regression algorithm. This disadvantage is minimized by the additionally included classifier in the compound model since it is trained with the true labels, LOS. LOS conveys the actual situation of direct or indirect line of sight. The use of the parameter $H_{B,AV}$ (that is the mean height of the buildings for a given coverage area) brings the advantage in predicting the attenuation of the proposed approach. The average height of buildings can be easily found or determined without an accurate 3D Geographic Information System (GIS). Furthermore, instead of the exact statistics for a given altitude profile, the model can use the average estimates of the other two input parameters, $H_{r,AV}$ and $H_{r,STD}$, in order to predict the coverage. In this case, it can be included in the group of zonal models and it will suffer their disadvantage—lesser accuracy.

For brevity, the output parameter (path loss) is denoted as *y*, i.e., y=L, where the true value for the path loss is calculated according to the radio link budget formula:

(9) $$y=L\;=P_{T}\;\left[\mathrm{dBm}\right]+G_{S}\;\left[\mathrm{dBi}\right]+G_{M}\;\left[\mathrm{dBi}\right]-P_{R}\;\left[\mathrm{dBm}\right],$$

where $P_{T}$ is the transmitter power, $P_{R}$ is the received power, and $G_{S}$ and $G_{M}$ are the antenna gains.

### 2.3. Architectures of the Individual Models

**Figure 2:**
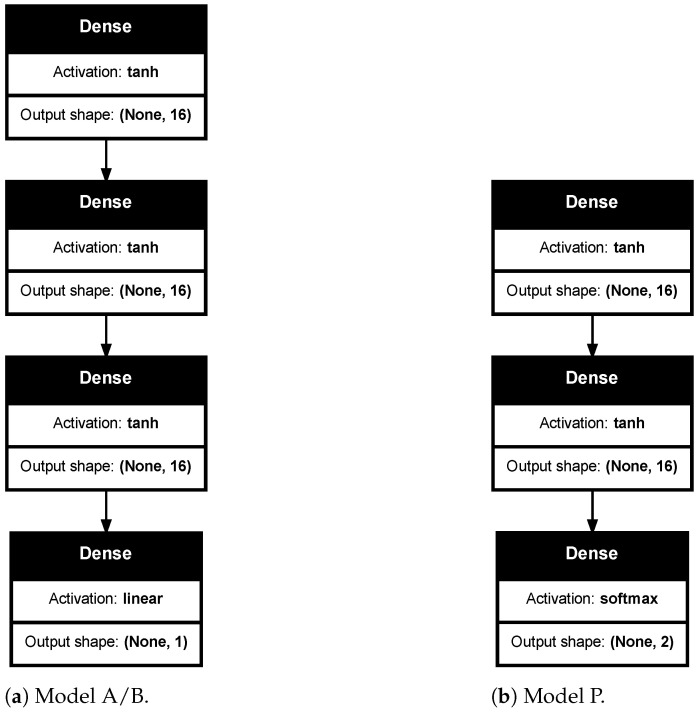
Architectures of the proposed individual models (**a**,**b**).

In Figure 2, the architectures of the individual models used are presented. The regression models (Model A and Model B) share a similar structure (Figure 2a). It consists of three hidden fully connected layers (Dense) with 16 perceptrons [24] in each. A fully connected layer means every input of the input vector influences every output of the output vector (all possible connections layer-to-layer are present). The output shapes of each layer are denoted with pairs enclosed in parentheses, where “None” indicates a dummy dimension. The input shape of each layer is the output shape of the previous one, except for the first layer whose inputs are equal to the dimension of x. The used activation function for all layers except the last one is tanh [25]. The output layer has a linear activation function, which is well suited for regression problems.

Models A and B are prone to over-fitting. This can be prevented using kernel regularization by adding penalty factors to the layers. In our case, L2 kernel regularization is implemented [26] in each hidden layer by modifying the cost function, *C* [26]:

(10) $$C=\sum_{i=1}^{N}{\left(y_{i}-f\left(x_{i}\right)\right)}^{2}+\lambda \sum_{j=1}^{M}w_{j}^{2},$$

where $w_{j}$ are the weights of the inputs $x_{j}$ and *f* denotes the transfer function of the neuron. The value of the regularization factor λ=0.075 is same for each layer.

The classification model (Figure 2b) has similar structure of its hidden layers, but without any kernel regularization. The activation function of the output layer is of type softmax [25], which has the property to normalize the output of a network to a probability distribution over predicted output classes.

The complexity of the models (in terms of number of layers and number of units in each of them) is determined experimentally. Starting from a simple one, the complexity is gradually increased until no significant improvement is obtained.

## 3. Experimental Results

### 3.1. Experimental Dataset

**Figure 3:**
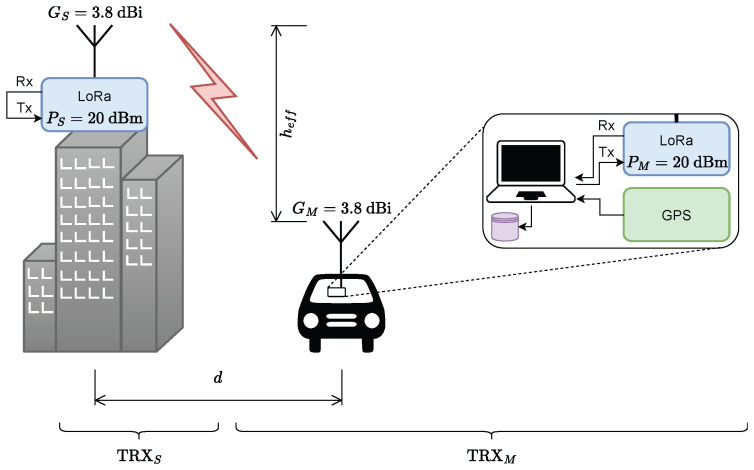
The measurement setup through which the experimental dataset is created.

The dataset for training the models and evaluating the performance of the proposed approach is provided by a dedicated measurement setup developed by the authors. The objective of the measurement is to create a dataset of primary parameters by which the attenuation can be determined as a function of transmitter and receiver locations, absolute altitudes of the transmitter and receiver antennas, and statistics of the profile transmitter–receiver. Because the operating frequency is in the free ISM range, LoRa modules for machine-to-machine communications are used as measurement transceivers. Each module consists of an ultra-low-power long-range transceiver of type SX1276 made by Semtech Corporation [27] and a microcontroller for control, processing, and interfacing via a universal asynchronous receiver–transmitter (UART). Another reason to use these modules is because the LoRa technology is tied to a high receiver sensitivity of −148 m. This implies measurements of greater distances between the transmitter and the receiver, especially in urban areas. Doppler shift is compensated for a frequency of up to 31.5 kHz at a bandwidth of 125 kHz. The operating frequency of 433 MHz allows ground mobile measurements with high offset rates. The software (version 1.0.0) for the embedded controller has the function to extract the received power value averaged over the received digital frame. In Figure 3 is depicted the proposed measurement concept as well, as the used equipment.

The stationary part, $TRX_{S}$, consists of a LoRa transceiver and J-pole antenna mounted at a suitable location on a building in the area under examination. The mobile part, $TRX_{M}$, also contains a LoRa transceiver and J-pole antenna, both of the same type as in the stationary subsystem. Additionally, there are a GPS receiver with a GPS antenna and a personal computer with installed and configured processing software. The LoRa transceiver and GPS receiver are connected to the computer via USB interface. The $TRX_{S}$ transceiver is configured in repeater mode. $TRX_{M}$ transmits a packet of data at a certain time interval. $TRX_{S}$ relays the received packet back to $TRX_{M}$. The receiver at $TRX_{M}$ processes the packet by measuring the received signal power, $P_{R,k}$. Here, *k* is the index associated with the *k*-th measurement point. For each measurement point *k*, the personal computer receives the measured power, $P_{R,k}$, from the LoRa module and associates it with the given GPS coordinates (latitude, longitude, and altitude), plus a timestamp. Such records are automatically saved to a database file.

**Figure 4:**
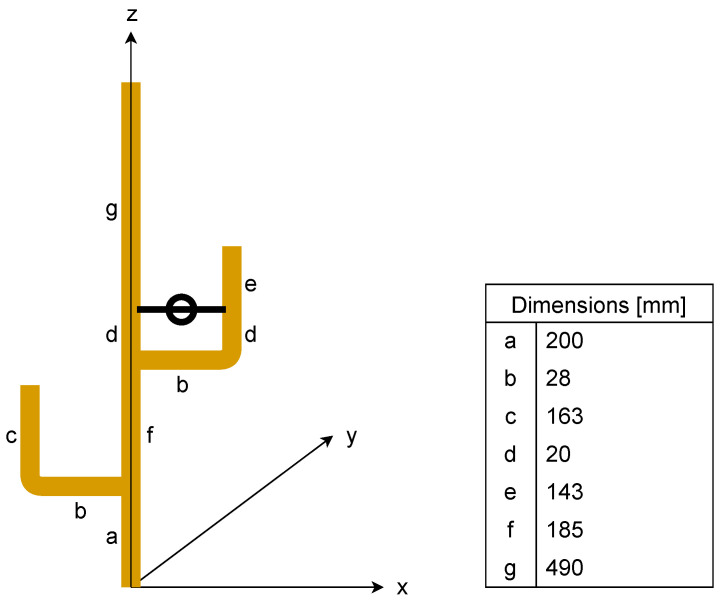
The used J-pole antenna. The length of each elements is given in the table.

The two nodes are equipped with identical J-pole antennas (Figure 4) with ground decoupling chokes [28,29].

The antennas operate on vertical polarization and provide omnidirectional patterns in the horizontal plane. They are made of copper pipes with a diameter of 6 mm. The ground decoupling choke provides cancellation of the currents in the mounting mast and the ground below (the car roof in the case of mobile applications), and therefore improves the pattern stability and noise temperature as well. An additional benefit of using the J-pole antenna (instead of the whip or monopole antennas, for example) is that the former cancels the unwanted out-of-band signals that can easily saturate the unprotected front-end of the LoRa receiver.

The operating parameters of the measurement system are transmitter output power $P_{T}=20\mathrm{dBm}$; antenna gain $G_{S}=G_{M}=3.8\mathrm{dBi}$; and operating frequency f=433MHz.

The areas for which the measurements are carried out are selected so that different relief types are presented. The selection includes rural, suburban, and urban areas with direct and indirect lines of sight at different heights of the stationary station antenna, geographical relief, and average height of buildings. The goal is to have a mixture of data through which the training model is adequate for each type of coverage. For example, Septemvri (Bulgaria) and Belogradchik (Bulgaria) are small towns with different terrain types. The measurement conditions and parameters for each selected area are summarized in Table 1.

**Figure 5:**
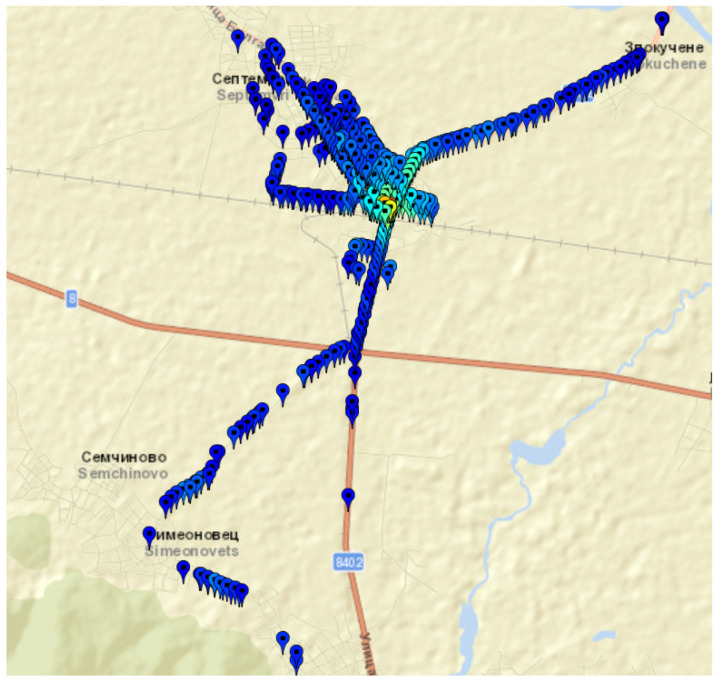
Map of geographical points associated with measurement records for Septemvri town (Bulgaria), rural and suburban areas.

**Figure 6:**
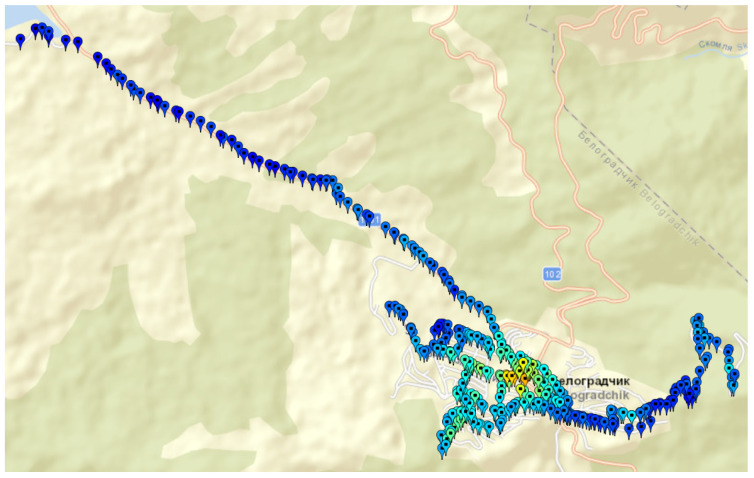
Map of geographical points associated with measurement records for Belogradchik town (Bulgaria), rural and suburban areas.

Ground mobile measurements at a low vehicle speed of no more than 30 km $h^{-1}$ are carried out for the selected areas. The mobile antenna is mounted on the top of the vehicle at a height $h_{M}=1.5$ m. The total number of measurement records is 4490. In Figure 5 and Figure 6 are shown the maps of geographical points associated with measurement records for Septemvri and Belogradchik.

**Figure 7:**
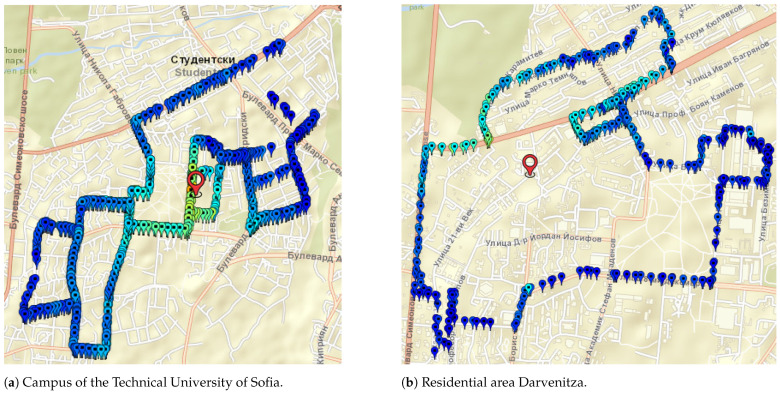
Map of geographical points associated with measurement records for two selected places in Sofia city (Bulgaria), urban and suburban areas.

Septemvri and Belogradchik are small towns, but the terrain is quite different. For these towns, the selected geographical points are both inside and outside the town, in order to cover rural coverage scenarios. For Septemvri, the antenna of the stationary station is above the average level of the rooftop buildings, while for Belogradchik it is on the terrace of a brick building. Most buildings are made of brick masonry. In Figure 7 are shown the maps of geographical points associated with measurement records of two areas in Sofia city (Bulgaria). The first is the campus of the Technical University of Sofia (Figure 7a). Residential area Darvenitza (Figure 7b) is the second one.

The campus of the Technical University of Sofia is located at the foot of Vitosha Mountain. The terrain is mixed—flat, mountain and foothill. Here, the measurements are performed at different heights of the antenna of the stationary station. The same procedure is followed for residential area Darvenitza.

### 3.2. Training and Validation

The parameters of all individual models are optimized using the gradient descent approach. This is achieved with the well-known back-propagation method [24].

For training, validation, and testing of each model, the available dataset is split randomly into two subsets. The first one is intended for training and validation, whereas the second is used for testing and performance evaluation. The proportion is 50% for training plus validation and 50% for testing. This spit leads to imbalanced data since the samples that are annotated as adequate for Model B are several times more than those for Model A. Because Model A and Model B preform regression, this imbalance is not crucial for their proper training. However, such disproportion is a significant concern for Model P fitting since its purpose is to perform classification. To overcome this problem, the training data for the minority class are up-sampled using the approach referred as the Synthetic Minority Oversampling Technique (SMOTE) [30]. This method inserts synthetic samples along the line between two data points in the minority class. This procedure is repeated until balanced data is ensured. SMOTE does not cause data loss and generally does not introduce over-fitting of the model.

In the training validation procedure, the k-fold method is applied for cross-validation of each model. The number of folds is selected as five, thus in each iteration the validation is with 20% of the data. The “best” fold is selected according to the minimal value of the loss function for the validation data. After that, the model is retrained with the selected subset, and finally its structure and weights are stored.

**Figure 8:**
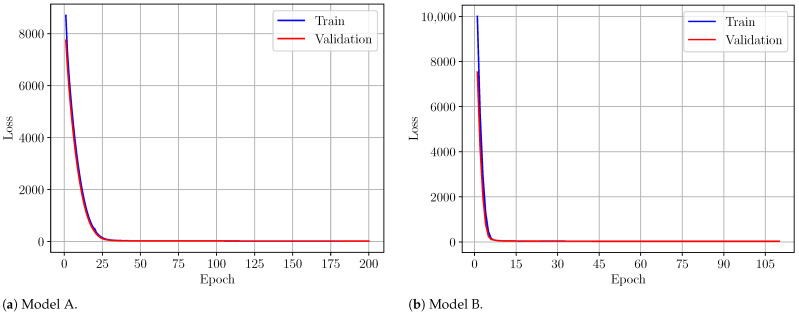
Loss function minimization during training of Models A/B, (**a**,**b**).

For Models A and B, the optimization is based on minimization of the mean squared error between the true output and predicted one. As can be seen from Figure 8, the training process of Models A and B is associated with good convergence of the loss function.

The maximum number of epochs is set to 200, but early stopping is allowed when no significant improvement is obtained on two consecutive steps.

Model P is trained according to loss function minimization, based on binary cross-entropy [31]:

(11) $$H_{p}\left(q\right)=-\frac{1}{N}\sum_{i=1}^{N}\left[y_{i}\log\left(p\left(y_{i}\right)\right)+\left(1-y_{i}\right)\log\left(1-p\left(y_{i}\right)\right)\right].$$

**Figure 9:**
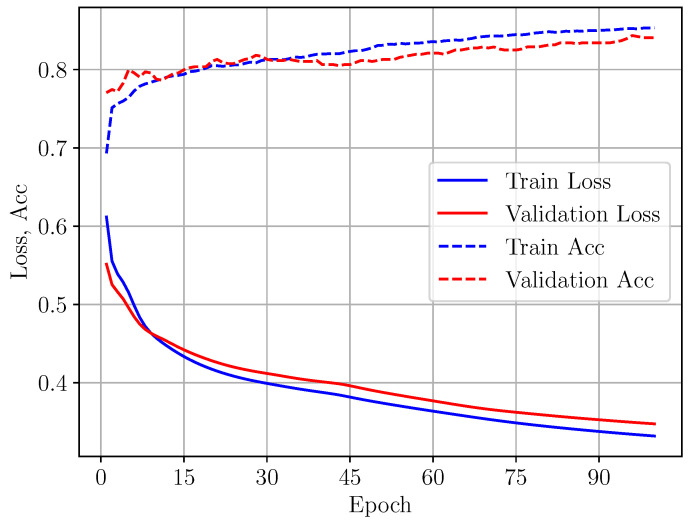
Loss and accuracy (Acc) curves improvement during training and validation of Model P.

This can be regarded as a measure for dissimilarity between the true labels and the predicted probabilities of inputs being in the positive class, where *y* is the output label and $p\left(y\right)$ is the predicted probability of the sample being 1 for all *N* samples. In Figure 9 is visualized the improvement of accuracy and loss functions during training of Model P.

As can be seen, the validation accuracy reaches nearly 85%.

### 3.3. Testing and Performance Evaluation

The performance of Model P is evaluated using the confusion matrix and Receiver Operating Curve (ROC), calculated when the model classifies the samples from the testing subset. ROC evaluates the performance of a model at all possible classification thresholds, showing the dependency between the true positive rate (TPR):

(12) $$TPR=\frac{TP}{TP+FN}$$

and false positive rate (FPR), defined as:

(13) $$FPR=\frac{FP}{FP+TN},$$

where TP is the true positives, FN is the false negatives, FP is the false positives, and TN denotes the true negatives of the classification. The area under the ROC curve, AUC, is a widely used estimate of the classification performance:

(14) $$AUC=\int_{0}^{1}TPR\left(FPR\right)dFPR.$$

**Figure 10:**
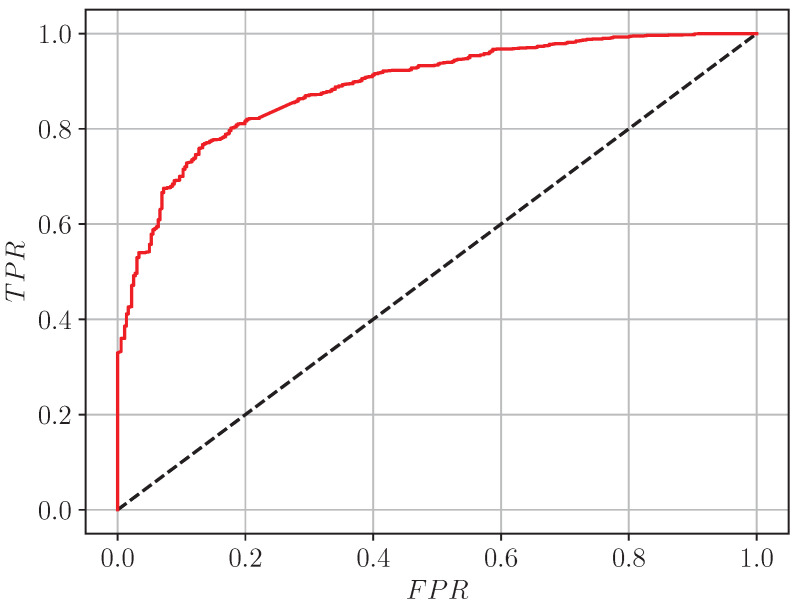
ROC curve of Model P (red line) with AUC=0.889. The random chance line is the black dashed one.

A perfect classifier has an AUC value equal to 1. In Figure 10 is shown the ROC curve of Model P (red line). The achieved value of the area under the ROC curve is AUC=0.889. The so-called random chance line (the black dashed one) is also given in the figure. It corresponds to a complete classification uncertainty. By visual inspection, the “elbow” of the curve does not lie on the diagonal $\left(FPR=0,TPR=1\right)\to \left(FPR=1,TPR=0\right)$. This corresponds to a decision threshold different from 0.5 for binary classification. This is an indicator that the model is not perfect. Nevertheless, considering the corresponding AUC, the model has a good discrimination capacity.

**Figure 11:**
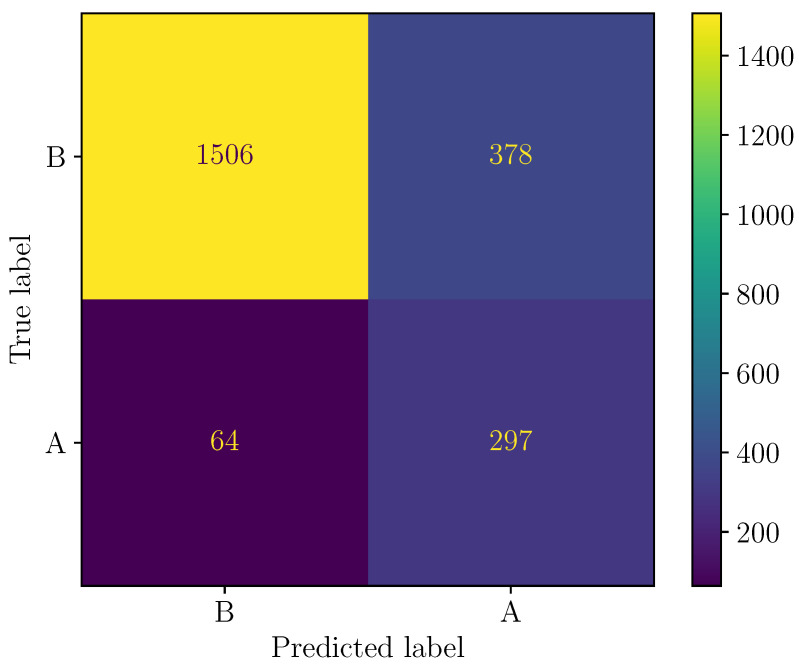
Confusion matrix of Model P (label “A” corresponds to LOS scenario, whereas “B” is for NLOS).

The confusion matrix is an another way to represent the performance of a classification model. The entries correspond to the number of true positives, false negatives, false positives, and true negatives. Figure 11 presents the confusion matrix when Model P is used to classify the samples from the testing dataset.

Considering entries of the confusion matrix, the accuracy of a model can be calculated according to:

(15) $$Acc=\frac{TP+TN}{TP+TN+FP+FN}.$$

For Model P, the achieved accuracy is Acc=80.3%.

**Figure 12:**
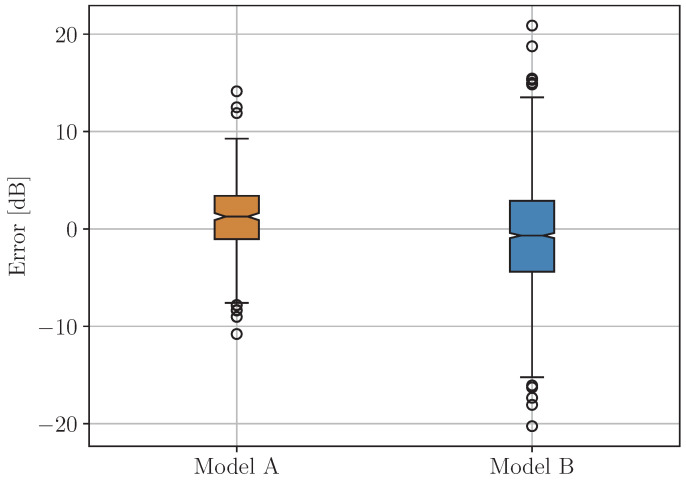
Boxplot diagrams of the prediction error of Models A and B.

Models A and B separately, as well as the compound one (with “soft” and “hard” combinations), are evaluated in terms of their performance using the error $e=y-\hat{y}$, where *y* is the true value and $\hat{y}$ is the predicted one. The performance of Models A and B is evaluated using only those samples from the testing subset that are adequate for the particular model. This is achieved with separation, based on the true labels. In Figure 12, diagrams of type boxplot are shown, visualizing the statistics of the prediction error of Models A and B.

**Figure 13:**
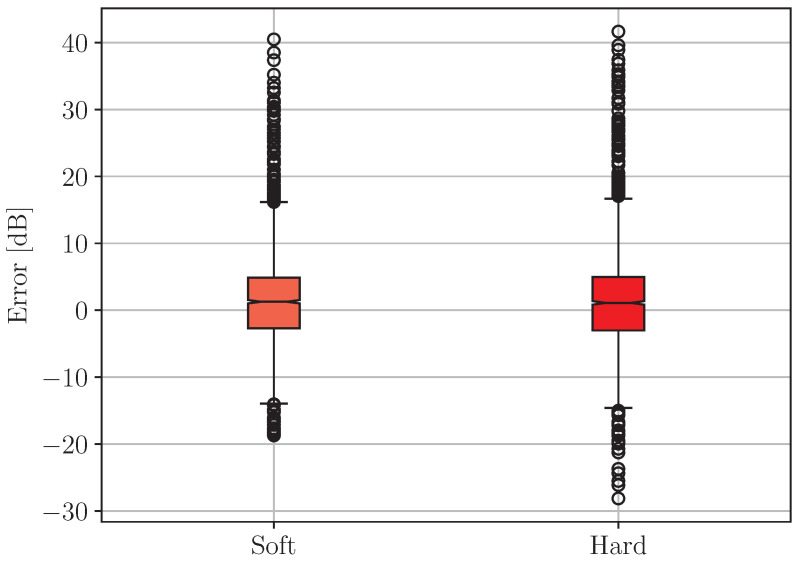
Boxplot diagrams of the prediction error of the compound model with “soft” and “hard” combinations of the outputs.

As can be seen, the error values of Model A are more tightly grouped than those associated with Model B. Similarly, the boxplot diagrams of the prediction error of the compound model are given in Figure 13 for “soft” and “hard” combination.

**Figure 14:**
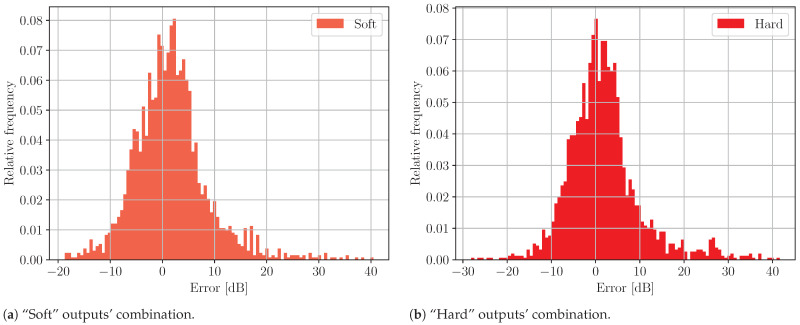
Histograms of the prediction error of the compound model with (**a**) “soft” and (**b**) “hard” combinations of the outputs. A normalization is made in respect of the total number of elements.

In the case of the compound model, the number of outliers is significantly higher than those presented for individual ones. The obvious reason for this is that the classification accuracy of Model P is far from perfect. In Figure 14, the corresponding histograms of the prediction error are shown.

No significant difference in terms of distribution type can be seen. The medians and interquartile ranges are also similar.

There are several numerical quantities by which the performance of a regression model can be evaluated. The first one is the coefficient of determination:

(16) $$R^{2}=1-\frac{\sum_{i=1}^{N}{\left(y_{i}-{\hat{y}}_{i}\right)}^{2}}{\sum_{i=1}^{N}{\left(y_{i}-\bar{y}\right)}^{2}},$$

where $\bar{y}=\frac{1}{N}\sum_{i=1}^{N}y_{i}$ is the mean of the true values and *N* is the number of data points. The coefficient of determination is a statistical measure of how well the predictions approximate the real data points. If $R^{2}$ is 1, this is an indication that the regression predictions perfectly fit the data. The second quantity is the well-known root mean square error:

(17) $$RMSE=\sqrt{\frac{1}{N}\sum_{i=1}^{N}{\left(y_{i}-{\hat{y}}_{i}\right)}^{2}}.$$

The third widely adopted indicator is the mean absolute error:

(18) $$MAE=\frac{1}{N}\sum_{i=1}^{N}\left|y_{i}-{\hat{y}}_{i}\right|.$$

For completeness, the mean value, $\mu_{e}$, and standard deviation, $\sigma_{e}$, of the error, *e*, are calculated as well. In Table 2 are summarized the quantities that characterize the performance of the compound model and individual ones.

As can be seen from Table 2, Model A is almost perfect. This can be expected since the path loss in LOS propagation conditions can be predicted with a high level of confidence. The compound model with a “soft” combination performs better than the “hard” type. Since the “hard” type has no advantages over the “soft” one, it can be rejected in future.

**Figure 15:**
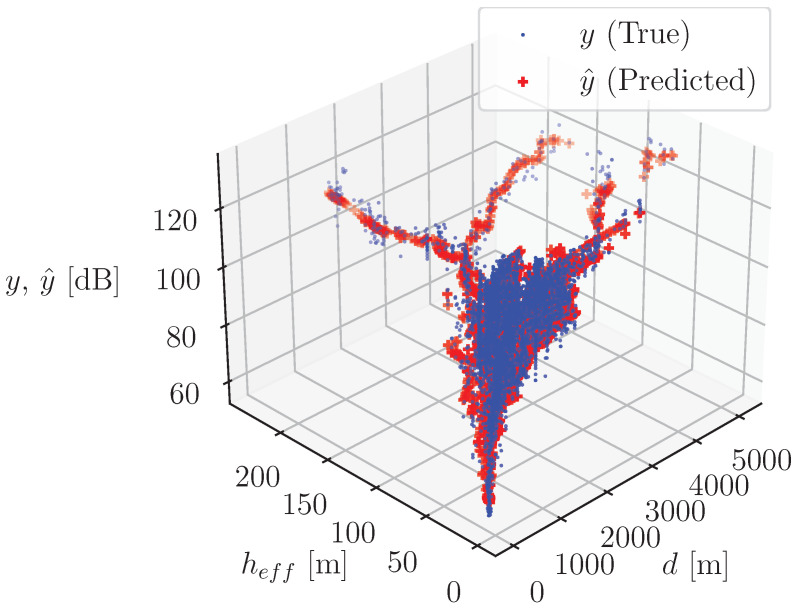
True and predicted outputs of the compound model with the “soft” combination versus distance, *d*, and effective antenna height, $h_{eff}$ (all samples from the dataset are used).

In Figure 15, the true and predicted outputs are visualized when using the compound model with the “soft” combination versus distance, *d*, and effective antenna height, $h_{eff}$.

All available samples from the dataset are used to produce these predictions. These results confirm the achievement of good balance in terms of low prediction error and generalization capability.

## 4. Discussion

More accurate conclusions about the achieved results can be made when comparing the performance of the proposed approach with performances reported in other competitive studies. At the moment, there are no published studies that refer to the same conditions and limitations (operating frequency, area type, etc.). Such comparisons would not be entirely correct because it cannot be said that if a model has a low value of RMSE or $R^{2}$ among those compared it is the best one. Accuracy depends on the choice of input parameters, the conditions under which they are measured, the accuracy of the measurements, and the regression algorithms used. The models that demonstrate high accuracy are valid only for a certain type of terrain, for a specific antenna height, and for certain propagation conditions—LOS/NLOS scenarios. The dispersion of the large-scale fading, which directly affects the accuracy of the model, depends on the type of terrain and the type of environment: rural, urban, sub-urban, etc. Using measured data from environments with a larger fading dispersion will also lead to larger model error, despite the applied machine learning algorithm. If, when comparing two models, one of them has a slightly worse performance, but it is evaluated under more severe conditions (area type, LOS/NLOS scenarios, etc.), then this model would have more applicability. Considering these stipulations, a conditional comparison in terms of achieved performance is given in Table 3. The goal here is not to make an exact performance comparison, but rather to evaluate how competitive the proposed approach is in terms of good balance between accuracy and generality.

As can be seen, the proposed approach has an RMSE that is comparable or even better than that reported in studies under similar conditions [11,32]. In terms of $R^{2}$ and MAE, both [11,32] are outperformed. The approaches proposed in [16,17] have better values of RMSE and $R^{2}$, but they are intended for urban areas only. The RMSE achieved in [32] appears to be slightly better, but the operating frequency is quite different. On the other hand, the cited study is applicable for suburban areas only.

Analyzing the numerical quantities that indicate the accuracy of the prediction and the results given in Table 3, the following conclusions can be drawn regarding the proposed approach:

- With five properly selected input parameters, the proposed compound model demonstrates satisfactory prediction performance (RMSE=7.3 dB and $R^{2}=0.702$) for its practical application. This is valid for different antenna heights, various area types (rural, suburban, and urban), and for both LOS/NLOS scenarios;
- With an appropriate combination of simplified ordinary neural structures with relatively small number of layers, a satisfactory prediction accuracy can be achieved that is comparable to the one reported in other similar studies;
- The two regression models also have high prediction accuracy (RMSE of 3.8 dB and 5.3 dB). These values of the RMSE are comparable to those reported in [33]. The models can be used separately when LOS/NLOS scenarios are predetermined;
- The used input parameters are easy to obtain and calculate;
- The achieved results are characterized with a high degree of confidence, considering the size and representativeness of the dataset (nearly 4500 measurement records for urban, suburban, and rural areas);
- The binary classifier is the bottleneck of the compound model’s performance. If this classifier is refined, the predictive accuracy will approach that of the individual regression models.

The achieved accuracy of the regression models is entrusted within the limits of variation of the input parameters and the environment in which their values are measured. The neural networks are trained under these conditions also. The estimates of the accuracy of the compound model and the individual ones are guaranteed under the following restrictive conditions:

- 2D distance between antennas: d>10 m;
- Effective antenna height: $0\;m\leq h_{eff}\leq 237\;m$;
- Urban/suburban/rural environments;
- Brick and reinforced concrete buildings in urban and suburban areas;
- Flat/foothill/mountain/hilly relief types;
- Areas with humid continental climate;
- Operating frequency: f=433 MHz.

The proposed approach can be used to create other regression models of path loss prediction under other boundaries and conditions beyond those mentioned above. Its generalization consists in the fact that it can be used to create a model summarizing the different types of areas: rural/suburban/urban, and for LOS/NLOS propagation conditions. The model is not bound to specific values of antenna heights. From another point of view, its generalization is also expressed in the fact that the model can be easily transformed from a point-to-point into an area-to-area type by replacing the last two input parameters with their average values for a given terrain type.

## 5. Conclusions

A machine learning approach for path loss prediction is presented in this study. A compound architecture is proposed, by which low prediction error is achieved for various propagation conditions.

The selection of the input parameters of the model is performed not only on the basis of the physical processes for the propagation of the electromagnetic wave, but also on the indirect statistical characteristics related to the terrain morphology. Through them, the proposed model indirectly accounts for diffractive propagation properties and path loss from large-scale fading. The variety of measured input parameters at different antenna heights and types of areas allows an adequate model to be synthesized with automatic classification and consideration of direct and indirect line-of-sight scenarios, applicable to urban, suburban, and rural areas. Another advantage of the model is that, when terrain statistics are missing, they can be replaced with averages, typical for the given coverage area. If these are applied, then the model turns into zonal. The prediction accuracy will be lower in this case.

The obtained experimental results show excellent performance of the compound model in terms of a root mean square error of the prediction as low as 7.3 dB and a coefficient of determination as high as 0.702. This accuracy is fully satisfactory for its practical application. Moreover, the accuracy can be further increased if an improved version of the classification model is developed. This is because the two regression models are characterized by even better accuracy (root mean square error of the prediction of 3.8 dB for line-of-sight scenario and 5.3 dB for non-line-of-sight condition).

The proposed model can be trained and successfully applied for any operating frequency in the decimeter wavelength range and for other propagation environments and conditions.

## Figures and Tables

**Table 1:** Measurement conditions and parameters for each area.

Area	${\mathrm{TRX}}_{S}$ Coordinates	${\mathrm{TRX}}_{S}$ Antenna Height $h_{S}$ $\left[m\right]$	Area Type	Relief Type	Average Buildings’ Height $\left[m\right]$	Maximum Terrain Unevenness $\left[m\right]$	Maximum Measurement Distance $\left[m\right]$
Septemvri town Bulgaria	lat. 42.20447 lng. 24.13740 alt. 240 m	8 (mounted on the roof of a brick building)	Rural, suburban	Flat	7	95	4824
Belogradchik town, Bulgaria	lat. 43.62802 lng. 22.68377 alt. 500 m	8 (mounted on the 3rd floor of a brick building)	Rural, suburban	Hilly	16	240	5150
Sofia city, Bulgaria, Campus of the Technical University of Sofia	lat. 43.65518 lng. 23.35418 alt. 596 m	8.5, 12, 15.5, 19, 25.5 (mounted on the roof)	Suburban, urban	Flat, mountain, foothill	12	28	1784
Sofia city, Bulgaria, res. area Darvenitza	lat. 42.65676 lng. 23.34264 alt. 595 m	6.25, 9.75, 30	Urban, suburban	Flat, mountain, foothill	24	19	1152

**Table 2:** Performance of individual and compound models.

Model	$R^{2}$	RMSE	MAE	$\mu_{e}$	$\sigma_{e}$
Model A	0.930	3.8 dB (4.45%)	3.0 dB (3.33%)	1.0 dB (0.98%)	3.7 dB (4.34%)
Model B	0.739	5.3 dB (4.90%)	4.2 dB (3.77%)	−0.7 dB (−0.87%)	5.3 dB (4.82%)
Compound					
with “soft”					
combination	0.702	7.3 dB (6.87%)	5.2 dB (4.81%)	1.6 dB (1.29%)	7.1 dB (6.75%)
Compound					
with “hard”					
combination	0.604	8.4 dB (7.89%)	5.8 dB (5.36%)	1.8 dB (1.14%)	8.2 dB (7.75%)

**Table 3:** Performance of the proposed approach compared with other benchmark methods/approaches (the original precision of the quantities is preserved).

Method/Approach	$R^{2}$	RMSE	MAE
ANN, ensemble learning ^1^ [16]	0.8951	2.9416 dB	1.2753 dB
SVR ^2^ [17]	0.8528	2.1568 dB	—
ANN-RBF ^3^ [17]	0.8965	2.4967 dB	—
ANN-MLP ^4^ [11]	0.3975	7.876 dB	5.896 dB
ANN ^5^ [32]	0.4168	6.9344 dB	5.2745 dB
Proposed in this study ^6^	0.702	7.3 dB	5.2 dB

^1^ Urban area. Operating frequency 1800 MHz. ^2^ Urban area. Operating frequency—not specified. ^3^ Urban area. Operating frequency—not specified. ^4^ Suburban area. Operating frequency 450 MHz. ^5^ Urban/suburban/rural area. Operating frequency 950 MHz. ^6^ Urban/suburban/rural area. Operating frequency 433 MHz.

## Data Availability

The datasets containing the input data are publicly available at https://zenodo.org/uploads/13320273 (accessed on 5 September 2024), DOI:10.5281/zenodo.13320273. The source codes are available from the corresponding author upon reasonable request.

## Abbreviations

The following abbreviations are used in this manuscript:

IoT	Internet of Things
IoE	Internet of Everything
ML	Machine learning
MAC	Media access control
RLC	Radio link control
SVR	Support vector regression
RBF	Radial basis function
ANN	Artificial neural network
MLP	Multi-layer perceptron
GP	Gaussian process
ISM	Industrial, scientific, and medical
M2M	Machine-to-machine
LPWAN	Low-Power Wide-Area Network
LOS	Line-of-sight
NLOS	Non-line-of-sight
GIS	Geographic Information System
UART	Universal asynchronous receiver–transmitter
SMOTE	Synthetic Minority Oversampling Technique
ROC	Receiver operating curve
